# Development and evaluation of double gene transgenic cotton lines expressing Cry toxins for protection against chewing insect pests

**DOI:** 10.1038/s41598-019-48188-z

**Published:** 2019-08-13

**Authors:** Hamid Anees Siddiqui, Muhammad Asif, Shaheen Asad, Rubab Zahra Naqvi, Sobia Ajaz, Noroza Umer, Naveed Anjum, Imran Rauf, Muhammad Sarwar, Muhammad Arshad, Imran Amin, Muhammad Saeed, Zahid Mukhtar, Aftab Bashir, Shahid Mansoor

**Affiliations:** 1 Agricultural Biotechnology Division, National Institute for Biotechnology and Genetic Engineering (NIBGE), Faisalabad, Punjab Pakistan; 2 Pakistan Institute of Engineering and Applied Sciences, Nilore, Islamabad Pakistan; 3 Department of Biological Sciences, Forman Christian College, Lahore, Punjab Pakistan

**Keywords:** Molecular engineering in plants, Plant sciences

## Abstract

Cotton is the main fiber producing crop globally, with a significant impact on the economy of Pakistan. Bt cotton expressing a *Cry1Ac* gene is grown over a large area in Pakistan, however, there is a major concern that bollworms may develop resistance. Here we have used a durable resistance strategy against bollworms by developing a double gene construct containing *Cry1Ac* and *Cry2Ab* (pGA482-12R) for cotton transformation. Both Cry toxin genes have been cloned in the same T-DNA borders and transferred successfully into cotton via Agrobacterium-mediated transformation. Both genes are expressed in transgenic cotton plants and is likely to help breeders in developing new cotton cultivars by incorporating these genes in cotton lines having no Bt genes or expressing *Cry1Ac* gene (Mon 531). Positive transgenic cotton was identified by PCR using specific primers for the amplification of both *Cry1Ac* and *Cry2Ab* genes. Cry1Ac and Cry2Ab expression was confirmed with an immunostrip test and quantified using ELISA that showed significant spatio-temporal expression of Cry2Ab ranging from 3.28 to 7.72 µg/g of the tissue leaf. Insect bioassay with army worm (*Spodoptera litura*) was performed to check the efficacy of NIBGE (National Institute for Biotechnology and Genetic Engineering) double gene transgenic cotton plants and up to 93% insect mortality was observed.

## Introduction

Cotton (*Gossypium hirsutum*) is among the most valuable crops in the world because of its fiber producing quality. Though cotton is mainly grown because of lint but the cotton seed is also potentially valuable source of oil (18–24%) and protein (20–40%). Around the world, almost 350 million people are linked to cotton industry for employment. Production of cotton is liable to biotic stresses including insect pests attack as well as abiotic factors like temperature, drought and salinity. Chewing insect pests include pink bollworm (*Pectinophora gossypiella*), armyworm (*Spodoptera littoralis*), cotton bollworm (*Helicoverpa armigera*) and spotted bollworm (*Earias insulana*)^1,2^. Farmers use chemical insecticides for controlling this menace of insect pests, but most of the pesticides applied by farmers are carcinogenic and neurotoxic in nature and are harmful to humans and other beneficial organisms. In addition to the resistance development in target insects^3^ because of the continuous persistence, these insecticides pollute land and water resources^4^. As an alternative to synthetic pesticides, genetically modified (GM) crops expressing *Cry1Ac* and *Cry2Ab* genes from *Bacillus thuringiensis*, were developed and widely adopted by the farmers in the world^5^. The area under GM crops has been greatly increased from 90 million hectares in 2005, 170 million hectares in 2012^6^ to 189.8 million hectares globally by 2017^7^.

Bt cotton expressing *Cry1Ac* and *Cry2Ab* genes played an important role in sustaining the cotton production because of having an inbuilt protection mechanism against insect pests^8^. Bt cotton was formally approved for commercial cultivation in Pakistan during 2010. Although it has provided better protection against bollworms, however, most of the available Bt cotton varieties grown have relatively low expression of Cry1Ac and even appropriate refuge plans are not followed^9^ which, imposes high selection pressure in target insect pests to develop resistance against Bt toxins. Field-evolved resistance in pink bollworm has been reported in China and India^10^. Fluctuations in the expression of Bt genes in different parts of the plant have been already reported^11^ with the growing age of the plant^12^, environment^13^ and shows inconsistent efficacy towards insect pests through the season^9^.

Consistent expression of Bt protein is vastly indispensable especially in vulnerable parts of the plant during the growing season for sustainably controlling the insect pests^9^. Bt protein is shown to have higher expression levels early in the season as compared to the late season, where its expression declines even from the critical level of toxin expression^14^. Ovary, squares, boll rinds and maturing seeds are shown to have the lowest toxin expression^15^, and while these are the most vulnerable parts for bollworms attack especially pink bollworm (*P. gossypiella*) which mainly feeds on the flower and seeds.

The main objective of this study was the development of insect-resistant cotton having double gene construct harboring *Cry1Ac* and *Cry2Ab* genes, and evaluation of these double gene cotton lines for Bt gene expression in different parts of the plant including leaves, boll rind and developing seeds at different stages of plant growth.

## Results

### Development and characterization of transgenic Coker312 cotton

**Figure 1:**
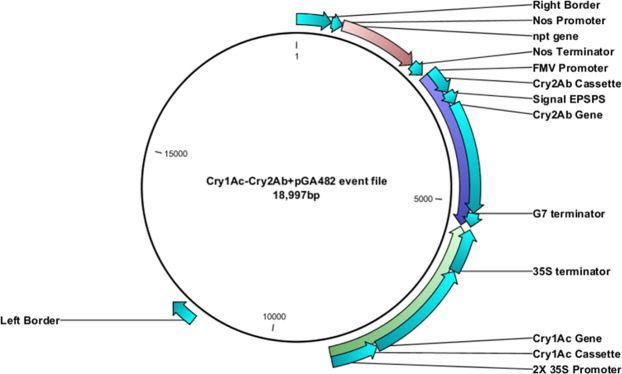
Map of the double gene construct having *Cry1Ac* and *Cry2Ab* in pGA482. *Cry1Ac* gene was cloned under 2 × 35S promoter and 35S terminator, while *Cry2Ab* was under FMV promoter and Nos terminator.

The double gene (*Cry1Ac* + *Cry2Ab*) construct was restricted with *Swa*I from a triple gene construct and successfully cloned into pGA482. The double gene construct in pGA482 has two adjacent cassettes. The size of the *Cry1Ac* cassette (2 × 35S- *Cry1Ac* -35S) was 3.5 kb, while the size of the *Cry2Ab* cassette (FMV-signal *EPSPS*- *Cry2Ab* -G7) was 2.8 kb (Fig. 1). The orientation of cassettes in pGA482 was confirmed through restriction analysis. The double gene construct was named as pGA482-*Cry1Ac*-*Cry2Ab* (pGA482-12R).

**Figure 2:**
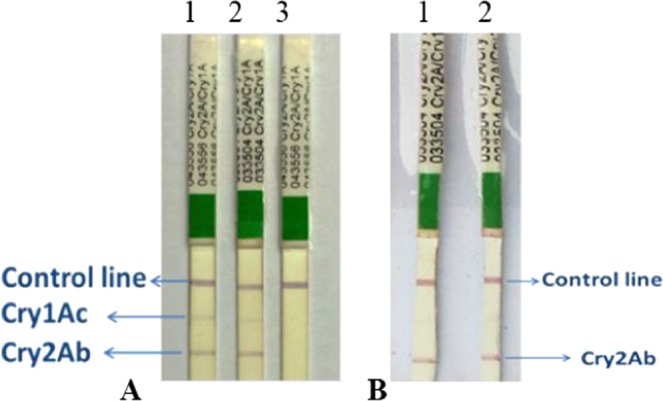
Immuno-Strip assay of NIBGE double gene cotton lines. (**A**) NIBGE cotton lines expressing Cry1Ac and Cry2Ab at 30 DAS. 1 was NIBGE-E36 cotton line and 2 was NIBGE-E45 cotton line and 3 was coker312 used as negative control. (**B**) Showing expression of Cry2Ab at 60 DAS and no expression of Cry1Ac. 1 was NIBGE-E36 and 2 was NIBGE-E45 cotton line.

Embryogenic calli of Coker312 cotton having the double gene construct (pGA482-12R) showed expression of Cry2Ab. Then from these calli, transgenic Coker312 cotton lines (NIBGE-E35, NIBGE-E36, NIBGE-E39, NIBGE-E45 and NIBGE-E49) were developed having pGA482-12R. These transgenic lines were then selected for further analysis through PCR using specific primers and PCR results showed amplification only with NIBGE specific primers (Tables 1 and 2). The immunostrip assay showed a very sharp band of Cry2Ab, whereas Cry1Ac band was very light as compared to Cry2Ab when tested at 30 DAS (Fig. 2A). The ELISA revealed similar results as the expression of Cry2Ab was up to 7.72 µg/g of leaf tissue. However, Cry1Ac expression was found very low (about 0.34 µg/g of the tissue). Cry2Ab expression was detected with an immunostrip assay at 60 DAS; however, the Cry1Ac expression could not be detected with the strip test at 60 DAS (Fig. 2A,B).

**Table 1:** Primer sequences for transgene analysis.

Sr. No.	Gene	Primer Name	Primer Sequence	ProductSize (bp)
1	*Cry1Ac*	CR1BDR5	ATGTCCATAAGGTGAGGTG	521
2		CR1BDF5	TTGCGTGAAGAGATGAGG	
3	*Cry2Ab*	CR2BDR4	ACTTGAGTGGCGTGTATG	614
4		CR2BDF4	CGGTGCTAACTTGTATGC	
5	*npt*II	APH2F	CTCACCTTGCTCCTGCCGAGA	215
6		APHR	CGCCTTGAGCCTGGCGAACAG

**Table 2:** Molecular analysis and evaluation of insect-resistant NIBGE double gene cotton lines.

Samples	Immuno-strip test		Insect bioassay	PCR analysis using specific primers					
	Cry1Ac	Cry2Ab	Armyworm %mortality	SadI	nptII	NIBGE Cry1Ac	NIBGE Cry2Ab	BG	BGII
NIBGE-E35	**+**	**+**	73	**+**	**+**	**+**	**+**	−	−
NIBGE-E36	**+**	**+**	93	**+**	**+**	**+**	**+**	−	−
NIBGE-E39	**+**	**+**	7	**+**	**+**	**+**	**+**	−	−
NIBGE-E45	**+**	**+**	80	**+**	**+**	**+**	**+**	−	−
NIBGE-E49	**+**	**+**	27	**+**	**+**	**+**	**+**	−	−
Coker312	−	−	0	**+**	−	−	−	−	−
BG	**+**	−	NT	**+**	**+**	−	−	**+**	−
BGII	**+**	**+**	NT	**+**	**+**	−	−	**+**	**+**

BG, MON531; BGII, MON15985; NT, not tested; (+), presence of gene and (−), absence of gene.

### Insect bioassay of NIBGE cotton lines

**Figure 3:**
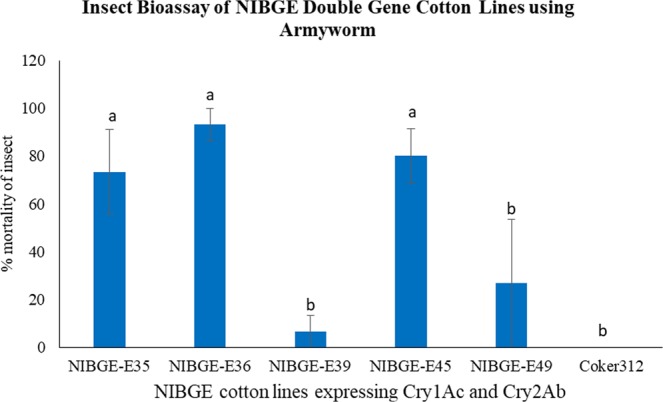
Insect bioassay of NIBGE double gene cotton plants using army worm *S. litura*. In insect bioassay of NIBGE double gene cotton plants, Coker312 was used as a negative control. %Mortality in insect bioassay of NIBGE double gene cotton plants vs control has been plotted and error bars represent the standard error among the replicates. Letters on the top of the bar indicate least significant difference between double gene transgenic cotton lines and negative control at P ≤ 0.05.

**Figure 4:**
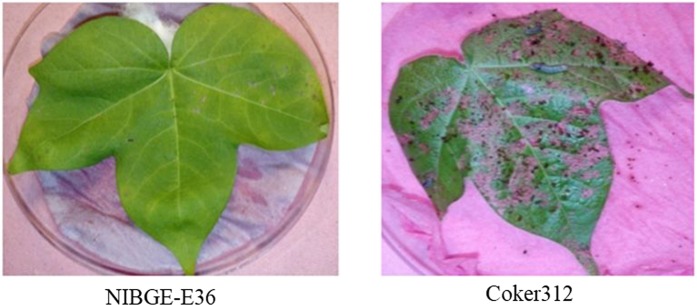
Leaf damage in insect bioassay of NIBGE double gene cotton plant (NIBGE-E36) vs Coker312.

Insect bioassay was carried out on leaves of NIBGE double gene transgenic cotton lines. Insect bioassays using transgenic cotton lines having the pGA482-12R double gene construct showed variation in insect mortality. Insect bioassay with laboratory-reared army worm (*S. littolaris*) was done. After 96 hours, there was up to 93% insect mortality on the NIBGE double gene cotton plants (Fig. 3 and 4; Table 2). There were significant variations among transgenic cotton lines and negative controls as statistically analyzed by ANOVA (p ≤ 0.0036).

### Spatial and temporal expression of Bt toxin

For the evaluation of spatial and temporal expression, leaves, bolls, and seeds from the double gene transgenic cotton lines were used to perform ELISA assay. Inter and intra plant variations of Cry2Ab expression was observed in NIBGE double gene cotton plants. Maximum Cry2Ab expression was observed in NIBGE-E45 cotton line at vegetative stage. Continuous decline of Cry2Ab expression was observed in NIBGE double gene cotton lines as the plants grow to maturity. In addition to the variations in temporal expression, intra plant variation has been observed. The results have shown variable expression of Cry2Ab in different parts of the plant, being maximum expression in the leaves 3.28–7.72 µg/g as shown in (Table 3) followed by maturing seeds 2.93–5.08 µg/g and the least expression was observed in boll rinds ranging from 0.1–0.6 µg/g of the tissue (Table 4).

**Table 3:** Variation in Cry2Ab expression in NIBGE double gene cotton lines with the growing season

DAS	NIBGE double gene cotton lines					Controls	
	NIBGE-E35	NIBGE-E36	NIBGE-E39	NIBGE-E45	NIBGE-E49	BG II	Coker312
30	6.51 ± 0.12	6.93 ± 0.54	4.45 ± 0.73	7.72 ± 0.44	6.14 ± 0.61	8.26 ± 0.43	0.05 ± 0
60	5.43 ± 0.23	5.72 ± 0.37	3.76 ± 0.79	6.96 ± 0.37	4.73 ± 0.46	6.83 ± 0.24	0.04 ± 0
90	3.96 ± 0.57	3.57 ± 0.23	3.28 ± 0.76	6.26 ± 0.15	4.75 ± 0.81	5.74 ± 1.52	0.08 ± 0.04

DAS, Days after sowing; BGII (MON15985) was used as positive control and non-GM Coker312 was used as negative control; ± , standard error.

**Table 4:** Expression analysis of Cry2Ab in NIBGE double gene cotton lines.

Sr. No.	Cotton lines	$$\begin{array}{c}{\bf{Cry2Ab}}\,{\bf{expression}}\,{\bf{in}}\,{\bf{boll}}\,{\bf{rinds}}\\ {\bf{Cry2Ab}}\,{\bf{Conc}}{\boldsymbol{.}}\,{\boldsymbol{\mu }}{\bf{g}}{\boldsymbol{/}}{\bf{g}}{\boldsymbol{(}}{\bf{ppm}}){\boldsymbol{\pm }}{\bf{SE}}\end{array}$$	$$\begin{array}{c}{\bf{Cry2Ab}}\,{\bf{expression}}\,{\bf{in}}\,{\bf{immature}}\,{\bf{seeds}}\\ {\bf{Cry2Ab}}\,{\bf{Conc}}{\boldsymbol{.}}\,{\boldsymbol{\mu }}{\bf{g}}{\boldsymbol{/}}{\bf{g}}\,{\boldsymbol{(}}{\bf{ppm}}{\boldsymbol{)}}{\boldsymbol{\pm }}{\bf{SE}}\end{array}$$
1	NIBGE-E35	0.2 ± 0.04	2.96 ± 0.96
2	NIBGE-E36	0.3 ± 0.08	5.08 ± 2.14
3	NIBGE-E39	0.5 ± 0.25	4.18 ± 1.87
4	NIBGE-E45	0.6 ± 0.22	3.05 ± 1.33
5	NIBGE-E49	0.1 ± 0.07	2.93 ± 0.82
6	BG II	4.0 ± 0.28	8.72 ± 1.21
7	Coker312	0.00	0.00

SE, Standard error.

## Discussion

To increase and prolong the efficacy of a single Bt toxin, multiple Bt genes with different mechanisms of action, have been introduced into various crops including cotton. *Cry2Ab* gene combined with *Cry1Ac* gene has been used to delay the development of resistance in target insects and provide synergistic effects against cotton bollworms^16^. However, the commercial events available from multinational companies use two separate events for incorporation into one cotton cultivar. Here we have used a strategy where two genes are cloned in the same T-DNA borders and were transformed into cotton. Here we have shown that both genes are expressed in transgenic cotton plants. The strategy is likely to help breeders in developing new cotton cultivars by incorporating these genes in cotton lines having no Bt genes or expressing *Cry1Ac* gene (Mon531 event).

The development of double gene Bt cotton may help to overcome resistance development in target insects against single-gene Bt cotton already commercialized in Pakistan. The development of resistance is an evolutionary process where insects develop resistance against the toxins that have been used to control those insects. Mutations detected in cadherin and ABC transporter genes are involved in resistance against Cry1Ac^10^ and have also contributed in the development of resistance in *Plutella xylostella* and *Trichoplusia ni*^17^. However, these mutations are not found to have cross-resistance against Cry2Ab^18^. Mutations in ATP-Binding Cassette (ABC) transporter gene has been found in resistance against Cry2Ab^19^. Bollworms having resistance against Cry1Ac can be controlled with Cry2Ab due to differences in their receptor binding sites. In the present scenario, cotton varieties harboring *Cry2Ab* gene will be a good option to control insect pests, particularly bollworms in a country like Pakistan where *Cry2Ab* has not been released commercially.

Bt cotton played a major role in increasing the production of cotton and reducing the use of insecticides in control of cotton bollworms. Expression of an adequate amount of Bt toxin is indispensable for sustainable insect pest control. It has been shown that the expression of Bt toxin in transgenic cotton declines with the passage of time over the growing season which imposes high selection pressure for the development of resistance in target insect pests. Similarly, resistance against Cry1Ac in *H. armigera* is also reported from northern China due to the mutations in cadherin gene^20,21^ and so far number of insect pests which, developed resistance against toxins have reached to 16 insect pests by 2016 from 3 insect pests in 2012^22^. Apart from the Cry1Ac toxin, Cry2Ab can also be considered as an alternative for controlling insect pests. Both toxins are different from each other in homology as well as in the mode of action, as Cry1Ac and Cry2Ab both involve different kind of binding receptors in the epithelial membrane of insect pests^23^. Binding of the toxin with the midgut receptors results in the formation of pores leading to the death of insect but pore formed in the epithelial membrane by the Cry1Ac is different than the pores formed by Cry2Ab^24,25^. Akhurst *et al*., (2003); Knowles and Dow (1993) observed Cry2Ab susceptible *H. armigera* strains that were resistant to Cry1Ac and hence, there is no involvement of cross-resistance^24,26^.

Over the growing season expression levels of Cry1Ac and Cry2Ab declines in various parts of the plant^15^ due to the various factors as Xia *et al*., (2005) suggested that decline in Cry1Ac expression could be due to the promoter methylation or post-translational gene silencing in later stages^14^ while Cry2Ab shows 10-fold higher expression than Cry1Ac^27^. In this study, Cry2Ab expression was measured in leaves, boll rinds, and seed through ELISA and a similar pattern of Cry2Ab expression was found with the lowest expression in boll rinds and highest in leaves as shown by^15^. Maturing seeds and flowers are the most vulnerable parts for bollworms attack especially pink bollworm, which imposes high selection pressure for the development of resistance in pink bollworm due to the very low expression of Cry1Ac as already reported by Fabrick *et al*., (2014) in US and India^10^.

Our main objective of this study was the development of double gene insect resistant cotton lines containing Bt genes and to evaluate the spatio-temporal expression level of Cry1Ac and Cry2Ab. Relevant to the previous study our results also showed inter and intra plant variations of Cry2Ab expression^15^. Similarly our results also show that Cry toxin expression level declines with the age of plant and in comparison to fruiting parts like maturing seed, leaves show a higher level of expression^15,28–30^. In this study, Cry2Ab expression in leaves was in the range of 3.28–7.72 µg/g of the tissue leaf while in maturing seed it was found from 2.93–5.08 µg/g of the tissue (Table 3 & 4). In boll rind expression was observed in the range of 0.1–0.6 µg/g of the tissue, which was critically lowest in all three parts.

Understanding the mechanism of declined Bt expression with the passage of time is a rather complicated process. The declined expression is supposed to be because of many factors which involve the overexpression of transgene in the early stages of development which later on leads to the post-transcriptional gene regulation and results in gene silencing. The promoter also plays an important role in the declined expression of endotoxin at later stages as it gets methylated. Consistent level of expression is highly desired for delaying resistance in insect pests. An effort should be made to find new efficient promoters showing consistent endotoxin expression; especially tissue-specific promoters can play an important role in this regard. Seed-specific promoter can be used for high level of expression in maturing seeds as it can play a positive role in controlling pink bollworm which specifically feeds on flower and seed.

## Methods

### Double gene (*Cry1Ac* + *Cry2Ab*) construct development

A double gene (*Cry1Ac* + *Cry2Ab*) construct was developed from an already available triple gene (*Cry1Ac* + *Cry2Ab* + *EPSPS*) construct (GenBank accession No. KX880509) in a pSB187 vector^2^ having hygromycin selection. For ease in cotton transformation and tissue culture, the construct was re-cloned into a vector with the nptII gene for kanamycin selection. For this purpose, the pGA482 vector was selected because it has been successfully used for cotton transformation. The *Cry1Ac* + *Cry2Ab* (12 R) double gene cassette was restricted from the triple gene construct*Cry1Ac* + *Cry2Ab* + *EPSPS* + psb187 (12ER) with *Swa*1 (blunt end cutter) and pGA482 was restricted with Hpa1 (blunt end cutter). After cleaning up these products, a ligation reaction was performed followed by transformation in *E. coli* and spread on LB agar plates having kanamycin for selection of transformed colonies. A positive clone (pGA482-12R) was identified as giving the appropriate restriction pattern with *Hind*III and *Xho*I. Gene orientation was verified by restriction analysis. Confirmed clone (pGA482-12R) was transformed into *Agrobacterium tumefaciens* strain LBA4404. Insertion into *Agrobacterium* was confirmed by colony PCR using *Cry1Ac*, *Cry2Ab* and nptII primer sets^2^ (Table 1)

### Cotton transformation

*Agrobacterium* (LBA4404 strain) containing the double gene (*Cry1Ac* + *Cry2Ab*) construct (pGA482-12R plasmid) was used for transformation of Coker312 cotton. Hypocotyl sections were excised from approximately 10-day old cotton seedlings and placed with gentle shaking in *Agrobacterium* cell suspensions having the pGA482-12R plasmid. Hypocotyls were blot dried using filter papers and then for co-cultivation transferred to MS medium with incubation at 26 ± 2 °C in the dark for 2 days. Hypocotyls were then shifted to callus induction medium (MS1) on kanamycin (50 mg/L) and cefotaxime (200 mg/L) with incubation at 28 °C using 16 hrs light. On callus formation, calli were tested and shifted to MS medium with kanamycin. Surviving calli were placed onto the embryo maturation medium (MS2) with kanamycin. Mature embryos, were transferred to embryo germination medium (MS3). Germinated somatic embryos were placed in jars containing MS4 medium. Individual rooted plantlets were transferred to pots in sterile soil. T_0_ and T_1_ plants were self-pollinated to obtain progeny. Molecular analysis of T_0_ and T_1_ plants was conducted to confirm insertion.

### Molecular analysis of transgenic plants

Immunostrip tests (Cat No. AS012 LS, Envirologix, USA) were conducted on putative cotton transgenic plants at 30 and 60 days after sowing (DAS). Two leaf punches were independently collected and were ground in 300 µL of 1X extraction buffer according to the given protocol (Envirologix, USA). The mixture was centrifuged at 13,000 rpm for 1 min and the supernatant was collected in a separate sterile Eppendorf tube. Double gene (*Cry1Ac* and *Cry2Ab*) immunostrip was inserted in the solution and was allowed to stand at room temperature for 5–10 minutes to develop the required band. Plants expressing Cry1Ac and Cry2Ab were selected for further verification through PCR. DNA was isolated from putative transgenic cotton plants. Primers specific to NIBGE *Cry1Ac* and NIBGE *Cry2Ab* genes were used for PCR amplification. Monsanto event-specific primers were also used to check the presence or absence of those events in NIBGE cotton lines. The reaction mixture was prepared using Dream Taq Green Master Mix (Cat No. K1081, Thermo Fisher Scientific), 5 µL (50 ng/µL) cotton DNA, 1 µL (10 µM) of each gene-specific primer (Table 1) and deionized PCR water to make total reaction volume of 25 µL. A Bio-Rad PCR machine was used with PCR profile as initial denaturation at 95 °C for 5 min, then 40 cycles with denaturation at 95 °C for 1 min, annealing at 55 °C for 1 min and extension at 72 °C for 1 min, with a final extension at 72 °C for 10 min. Primer sequences of Sad1, nptII, MON531, MON15985, MON1445 were taken from the GMO Detection Method Database (GMDD; www.gmdd.shgmo.org) and published literature^31,32^.

### Quantification of Cry1Ac and Cry2Ab by ELISA

T_1_ seeds of five events of NIBGE double gene cotton were grown. Bollgard II (BGII, MON15985 event) and non-GM Coker312 were grown as positive and negative control respectively in the experiment. Positive plants from PCR and immunostrip analysis were selected for quantification of Bt gene expression in leaf, boll rinds and seeds at different stages using standard ELISA kit (Catalog no. AP 005, Envirologix,). About 20–25 mg tissue was weighed before homogenizing the leaf tissue in respective buffer provided in the kit. ELISA was performed according to the given protocol (Envirologix, USA) for the quantification of Cry1Ac and Cry2Ab expression that was shown in µg/g of tissue weight, through standard curve drawn by plotting OD values from calibrators.

### Insect bioassays

Efficacy of the NIBGE cotton lines having Cry1Ac and Cry2Ab toxins was evaluated through detached leaf bioassay of cotton plants with first instar armyworm (*S. littolaris*) larvae. Three leaves from each transgenic cotton line were collected and placed separately in Petri plates on a slightly moist filter paper. Each leaf was infested with five first instar larvae. These plates were kept at 25 ± 1 °C and 50–70% relative humidity. During the assay, non-GM Coker312 was used as negative control. By using stereo-microscope insect mortality data was recorded for five consecutive days after 24 h. The recorded data was used for the statistical analysis using ANOVA and means were compared using least significant difference (LSD) test.
